# SHP‐1 suppresses endotoxin‐induced uveitis by inhibiting the TAK1/JNK pathway

**DOI:** 10.1111/jcmm.15888

**Published:** 2020-11-18

**Authors:** Xiaonan Zhuang, Jun Ma, Sisi Xu, Zhongcui Sun, Rong Zhang, Meng Zhang, Gezhi Xu

**Affiliations:** ^1^ Department of Ophthalmology Eye & ENT Hospital Fudan University Shanghai China; ^2^ Eye Institute Eye & ENT Hospital Fudan University Shanghai China; ^3^ Shanghai Key Laboratory of Visual Impairment and Restoration Fudan University Shanghai China; ^4^ NHC Key Laboratory of Myopia Fudan University Shanghai China

**Keywords:** inflammation, JNK, LPS, Müller cells, SHP‐1

## Abstract

We investigated how Src‐homology 2‐domain phosphatase‐1 (SHP‐1) regulates the inflammatory response in endotoxin‐induced uveitis (EIU), and the signalling pathways involved. One week after intravitreal injection of short hairpin RNA targeting SHP‐1 or SHP‐1 overexpression lentivirus in rats, we induced ocular inflammation with an intravitreal injection of lipopolysaccharide (LPS). We then assessed the extent of inflammation and performed full‐field electroretinography. The concentrations and retinal expression of various inflammatory mediators were examined with enzyme‐linked immunosorbent assays and Western blotting, respectively. SHP‐1 overexpression and knockdown were induced in Müller cells to study the role of SHP‐1 in the LPS‐induced inflammatory response in vitro. Retinal SHP‐1 expression was up‐regulated by LPS. SHP‐1 knockdown exacerbated LPS‐induced retinal dysfunction and increased the levels of proinflammatory mediators in the retina, which was abrogated by a c‐Jun N‐terminal kinase (JNK) inhibitor (SP600125). SHP‐1 overexpression had the opposite effects. In Müller cells, the LPS‐induced inflammatory response was enhanced by SHP‐1 knockdown and suppressed by SHP‐1 overexpression. SHP‐1 negatively regulated the activation of the transforming growth factor‐β‐activated kinase‐1 (TAK1)/JNK pathway, but not the nuclear factor‐κB pathway. These results indicate that SHP‐1 represses EIU, at least in part, by inhibiting the TAK1/JNK pathway and suggest that SHP‐1 is a potential therapeutic target for uveitis.

## 1 INTRODUCTION

Uveitis is an ocular inflammatory disease that causes prolonged visual loss in two‐thirds of patients^1^ and is responsible for about 5%‐10% of all cases of visual loss worldwide.^2^ Non‐infectious uveitis, especially idiopathic anterior uveitis, is the commonest type of uveitis in developed countries, whereas infection is the leading cause of uveitis in developing countries.^3^ Inflammatory cytokines play pivotal roles in uveitis. The levels of interleukin (IL)‐6, IL‐8, chemokine ligand 2 and interferon‐γ were elevated in aqueous humour samples from patients with idiopathic uveitis, and the IL‐6 level was correlated with the number of infiltrating neutrophils.^4^ The increased activation of retinal Müller cells, which are specialized glial cells, is also observed in uveitis. These cells display up‐regulated glial fibrillary acidic protein and secrete proinflammatory cytokines.^5^ Uveitis is normally managed by systemic, topical or intraocular administration of corticosteroids.^6^ However, side effects include increased ocular pressure, increased risk of cataract and greater susceptibility to infection.^7^ Therefore, there is a need to develop alternatives to corticosteroids for the treatment of uveitis.

Rodent endotoxin‐induced uveitis (EIU) is a widely accepted animal model of human uveitis, especially acute‐onset uveitis.^8^ EIU is generally induced by injecting lipopolysaccharide (LPS) into the footpad or the vitreous body of the eye.^8^, ^9^ LPS increases vascular permeability and subsequent cellular exudates, the two main pathological characteristics of EIU.^10^ This model is also characterized by the up‐regulated expression of inflammatory mediators, particularly tumour necrosis factor‐α (TNF‐α), IL‐6, IL‐1β and monocyte chemoattractant protein‐1 (MCP‐1), which peaks at about 24 hours after the administration of LPS.^11^ It was recently demonstrated that Müller cells also respond to LPS with reactive gliosis and the production of inflammatory cytokines.^12^, ^13^ Investigating the regulation of Müller cells during EIU may provide insight into strategies to control or treat ocular inflammation. Therefore, we used Müller cells to study the role of Src‐homology 2‐domain phosphatase‐1 (SHP‐1) in the retina.

Src‐homology 2‐domain phosphatase‐1, also known as protein‐tyrosine phosphatase non‐receptor 6, negatively regulates signal transduction pathways in the immune system by suppressing the downstream nuclear factor‐κB (NF‐κB) and mitogen‐activated protein kinase (MAPK) pathways.^14^ SHP‐1 is also in control of endotoxemia.^15^ Microglia from SHP‐1‐deficient motheaten mice produced more nitric oxide, IL‐1β, and TNF‐α in the central nervous system in response to LPS than the microglia from wild‐type mice.^16^ The expression of SHP‐1 was elevated in reactive astrocytes after focal cerebral ischaemia and might restrict reactive gliosis and inflammation.^17^ Retinal SHP‐1 expression has been confirmed in mice and is linked to progressive retinal degeneration.^18^ However, how SHP‐1 functions in retinal inflammation and reactive gliosis in Müller cells are not yet clear.

In this study, we investigated the role of SHP‐1 in EIU in vivo and in Müller cells in vitro to identify the signalling pathways involved. We found that SHP‐1 expression increased in the retina in response to EIU and in LPS‐treated Müller cells in vitro. SHP‐1 knockdown exacerbated retinal inflammation, whereas SHP‐1 overexpression weakened the inflammatory response. Finally, we demonstrated that SHP‐1 negatively regulates the inflammatory response by repressing the transforming growth factor‐β‐activated kinase‐1 (TAK1)/c‐Jun N‐terminal kinase (JNK) pathway.

## 2 MATERIALS AND METHODS

### 2.1 Animals

All animal experiments were approved by the Animal Ethics Committee of the Eye and Ear Nose Throat Hospital of Fudan University, China, and conformed to the ARVO Statement on the Use of Animals in Research. Six‐week‐old male Sprague‐Dawley rats were purchased from Slac Laboratories. The rats, weighing 160‐180 g, were kept in comfortable cages under a 12/12 hour light/dark cycle and were provided with ample water and food.

### 2.2 Intravitreal administration of lentivirus and EIU induction

Anaesthesia in rats was achieved by an intraperitoneal injection of ketamine (80 mg/kg), mixed with xylazine (10 mg/kg). SHP‐1 knockdown was achieved by the administration of a short hairpin RNA targeting SHP‐1 recombinant lentivirus (shRNA‐SHP‐1‐rLV; Genomeditech). The recombinant lentivirus encoding SHP‐1 (SHP‐1‐rLV; Genomeditech) was used to overexpress SHP‐1. Briefly, 3 μL of shRNA‐SHP‐1‐rLV or SHP‐1‐rLV (5 × 10^8^ transducing units/mL) was injected intravitreally into the right eye of each rat. As a control, 3 μL of blank‐recombinant lentivirus (blank‐rLV) was injected into the left eye. One week after transfection, the rats were intravitreally injected with 500 ng of LPS from *Escherichia coli* O111:B4 (Sigma‐Aldrich) diluted in 2 μL of phosphate‐buffered saline (PBS) to establish EIU or with 2 µL of PBS as the control. All intravitreal injections were performed with a microinjector (Hamilton).

### 2.3 Intravitreal administration of JNK inhibitor and LPS

SP600125 (Beyotime), an inhibitor of JNK phosphorylation, was first dissolved in dimethyl sulfoxide (DMSO; Sigma‐Aldrich) and then diluted with PBS to a final concentration of 0.1 mmol/L with 1% DMSO. The Rats in the blank‐rLV + PBS group received an intravitreal injection of 2 μL of PBS with 1% DMSO. The Rats in the blank‐rLV + LPS group were intravitreally injected with 500 ng of LPS diluted in 2 μL of PBS with 1% DMSO, whereas those in the blank‐rLV + LPS+SP600125 group or shRNA‐SHP‐1‐rLV + LPS+SP600125 group received 500 ng of LPS mixed with 0.2 nmol SP600125 in 2 μL of PBS with 1% DMSO.

### 2.4 Assessment of EIU severity

The severity of EIU in the anterior segment was assessed with biomicroscopy at 0, 24, 48, and 72 hours after the induction of EIU and was ranked with a previously reported scoring system,^19^ based on iris hyperaemia, the presence of exudate in the anterior chamber, hypopyon and pupil miosis.

### 2.5 Electroretinography

At 24 hours after the injection of LPS and after adaptation in the dark for 12 hours, the rats underwent electroretinography (ERG). The rats were anaesthetized as described above, followed by topical use of atropine sulphate, oxybuprocaine (Santen Pharmaceutical) and carbomer eye gel (Bausch & Lomb) for mydriasis, corneal anaesthesia and corneal hydration, respectively. The contact electrodes were placed on the central cornea, and two needle electrodes were placed subcutaneously near the nose and tail to act as the reference and ground electrodes, respectively. A visual electrophysiology system (Espion E3; Diagnosys UK) was used to record the maximum ERGs following a stimulus of 20 cds/m^2^ at a single pulse of 0.1 Hz in a completely dark background. The a wave amplitude was measured as the difference of amplitude between the baseline and the trough of the negative deflection. The b wave amplitude was measured from the trough of the a wave to the peak of the b wave. The ERGs were recorded 10 times per eye, and the mean value for each eye was calculated.

### 2.6 Collection of aqueous humour samples for enzyme‐linked immunosorbent assays (ELISAs) and cell counting

The aqueous humour collected from one eye of a rat represented one sample and five samples per group were used to measure the concentrations of specific inflammatory cytokines. The aqueous humour was collected from the anterior chamber with a microsyringe. The samples were kept on ice and then centrifuged at 300 *g* for 5 minutes. After centrifugation, the supernatant was used to measure the concentrations of IL‐6, IL‐1β, MCP‐1 and chemokine (C‐X‐C motif) ligand 1 (CXCL1) with ELISA kits (MultiSciences Biotech).The cell deposit obtained by centrifugation was resuspended in a volume of PBS identical to the amount of aqueous humour obtained from the eye. The number of free cells was then determined with a haemocytometer by two observers blinded to the experimental groups.

### 2.7 Preparation of paraffin‐embedded retinal sections for haematoxylin and eosin staining

The enucleated eyes were kept in 4% paraformaldehyde for 48 hours at room temperature and then embedded in paraffin. Sagittal sections (5 μm thick) from beneath the optic nerve were prepared and stained with haematoxylin and eosin (H&E). The sections were observed with light microscopy (Leica Microsystems). The numbers of cells that had infiltrated the anterior chamber and the vitreous body were counted by two observers blinded to the experimental groups.

### 2.8 Immunohistochemistry

The eyecups were fixed in 4% paraformaldehyde for 1 hour and dehydrated sequentially in 20% and 30% sucrose solutions at 4°C for 1 hour. After the anterior sections had been removed and the cups filled with optimal cutting temperature compound (Tissue‐Tek; Ted Pella), they were then frozen at −80°C, and cut in the sagittal direction (8‐μm‐ thick sections). After being blocked with 5% goat serum and permeated with 0.15% Triton X‐100 in PBS for 45 minutes, the sections were incubated with the following primary antibodies overnight at 4°C: mouse anti‐SHP‐1(sc‐7289, diluted 1:50; Santa Cruz Biotechnology); mouse anti‐pJNK (9255S, diluted 1:100; Cell Signaling Technology) and rabbit anti‐ glutamate synthase (GS) (ab49873, diluted 1:5000; Abcam). The sections were rinsed three times with PBS and incubated with a secondary antibody (A28180; Invitrogen; or 4414, Cell Signaling Technology) for 1 hour at room temperature. After the sections were rinsed three times, they were counterstained with Fluoroshield with DAPI mounting medium (ab104139; Abcam). The sections were then observed with a laser confocal microscope (Leica Microsystems).The immunofluorescence intensity in the images was measured with ImageJ software (National Institutes of Health).

### 2.9 Cell culture, transfection and LPS administration

The transformed rat retinal Müller cell line (rMC‐1) was a kind gift from Shanghai Tenth People's Hospital, Tongji University (Shanghai, China). The cells were cultured in low‐glucose Dulbecco's modified Eagle's medium (DMEM) supplemented with 10% foetal bovine serum and 1% penicillin/streptomycin at 37°C under 5% CO_2_ in a humidified incubator. The rMC‐1 cells were then transfected with blank‐rLV, SHP‐1‐rLV, or shRNA‐SHP‐1‐rLV. The efficiency of SHP‐1 overexpression or knockdown was estimated with Western blotting of SHP‐1. After stable transfection, the cells were seeded in six‐well plates and cultured for 12 hours before the LPS treatment. To assess the phosphorylation status of the target proteins, the cells were serum‐starved overnight before treatment. LPS was stored at a concentration of 1 mg/mL and added to the culture medium to achieve the appropriate final concentrations. After incubation for specific times, the cells were harvested for Western blotting.

### 2.10 Western blotting

The retina and cultured rMC‐1cells were homogenized in RIPA buffer (Beyotime), freshly mixed with phenylmethylsulfonyl fluoride, and lysed on ice for 30 minutes (rat retina) or 5 minutes (rMC‐1 cells). The lysate was centrifuged at 13 600 *g* for 5 minutes at 4°C; resuspended in 5× sample loading buffer (Beyotime) and boiled at 95°C for 5 minutes. The proteins were separated by sodium dodecyl sulphate‐polyacrylamide gel electrophoresis in 12% gel. The proteins were then transferred to a polyvinylidene difluoride membrane (Millipore), which was then blocked with 5% milk for 1 hour and incubated with diluted primary antibodies at 4°C overnight: rabbit anti‐SHP‐1 (ab32559, diluted 1:1000; Abcam); rabbit anti‐cyclooxygenase‐2 (COX2; ab15191, diluted 1:1000; Abcam); rabbit anti‐IL‐1β (AB1832P, diluted 1:1000; Millipore); rabbit anti‐macrophage inflammatory protein‐1α (MIP‐1α) (80044‐T32, diluted 1:800; Sino Biological); mouse anti‐MCP‐1 (66272‐1‐lg, diluted 1:800; Proteintech); rabbit anti‐NF‐κB p65 (4764S, diluted 1:1000; Cell Signaling Technology); rabbit anti‐NF‐κB phospho‐65 (3033S, diluted 1:1000; Cell Signaling Technology); rabbit anti‐SAPK/JNK (9252T, diluted 1:1000; Cell Signaling Technology); rabbit anti‐phospho‐SAPK/JNK (9251S, diluted 1:1000; Cell Signaling Technology); rabbit anti‐phospho‐TAK1 (AF3019, diluted 1:1000; Affinity Biosciences); and horseradish‐peroxidase (HRP)‐conjugated anti‐β‐actin (5125S, diluted 1:2000; Cell Signaling Technology). After the membrane was rinsed three times, it was incubated with the appropriate HRP‐conjugated secondary antibody (Millipore) for 50 minutes at room temperature. The membrane was rinsed three times, and the protein bands were detected with enhanced chemiluminescence fluid (Millipore). Images were taken with Kodak Image Station 4000MMPRO (Carestream).

### 2.11 Statistical analysis

Data were analysed with SPSS for Windows version 17.0 (IBM‐SPSS). The data were presented as the means ± standard deviations of at least three independent experiments. The number of individuals included in each group and the specific method used is stated in the figure legends. Differences were deemed statistically significant at *P* < .05.

## 3 RESULTS

### 3.1 LPS up‐regulates the retinal expression of SHP‐1 and SHP‐1 knockdown exacerbates LPS‐induced inflammation

**FIGURE 1:**
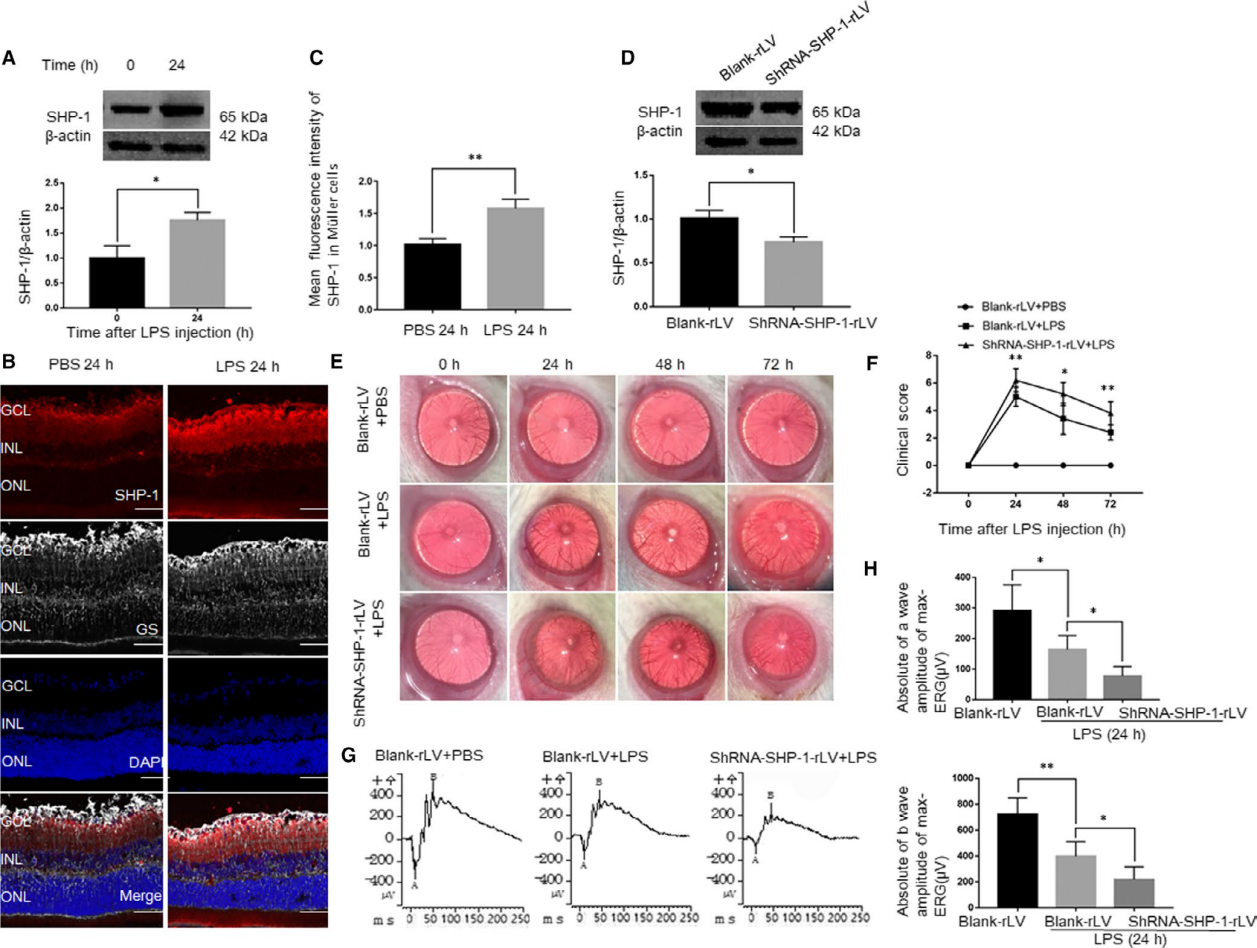
SHP‐1 is up‐regulated in the retina and Müller cells in EIU, whereas SHP‐1 knockdown aggravates inflammation and retinal dysfunction. A, Western blotting analysis of retinal SHP‐1 protein expression before and 24 h after intravitreal injection of LPS. Unpaired *t* test was used. n = 3 per group. B, Co‐immunofluorescent staining for SHP‐1 and glutamate synthase (GS) in the retina. Red: SHP‐1; grey: GS; blue: DAPI. Scale bar: 50 μm. GCL: ganglion cell layer; INL: inner nuclear layer; ONL: outer nuclear layer. C, Quantitative analysis of mean fluorescence intensities of SHP‐1 at sites of GS staining. Unpaired *t* test was used. n = 3 per group. D, Western blotting analysis of SHP‐1 expression 1 wk after intravitreal injection of blank‐rLV or shRNA‐SHP‐1‐rLV. Unpaired *t* test was used. n = 3 per group. E, Representative biomicroscopic images of the blank‐rLV + PBS, blank‐rLV + LPS and shRNA‐SHP‐1‐rLV + LPS groups at 0, 24, 48 and 72 h after LPS administration. F, Comparison of clinical scores, derived from biomicroscopic images, among the three groups. One‐way ANOVA followed by Dunnett's test was used. n = 5 per group. G, Representative maximum ERG traces recorded for the blank‐rLV + PBS, blank‐rLV + LPS and shRNA‐SHP‐1‐rLV + LPS groups at 24 h after LPS administration. H, Comparison of the amplitudes of a and b waves of maximum ERG tests in the groups mentioned above. One‐way ANOVA followed by Dunnett's test was used. n = 5 for the blank‐rLV + PBS group, n = 6 for the other groups.*P < .05 and **P < .01

Previous studies have shown that LPS‐induced inflammation is most severe about 24 hours after the administration of LPS and that the retina is particularly vulnerable to inflammation.^20^ Therefore, we chose this time to evaluate the role of SHP‐1 in the retina. We first determined the retinal expression of SHP‐1 in the EIU model and found that its expression increased by about 1.8‐fold at 24 hours after the administration of LPS (Figure 1A). To identify the localization of SHP‐1 in the retina after LPS treatment, we performed the immunofluorescent staining for SHP‐1 and GS (a marker of Müller cells). SHP‐1 expression was significantly up‐regulated in the endfeet of the Müller cells 24 hours after LPS treatment (Figure 1B,C). These results imply that SHP‐1 in Müller cells participates in the regulation of inflammation in the EIU model.

To determine the roles of SHP‐1 in LPS‐induced retinal inflammation, we knocked down retinal SHP‐1 and ascertained the knockdown efficiency with Western blotting (Figure 1D). After the successful knockdown of retinal SHP‐1, we replicated the EIU model in eyes previously transfected with blank‐rLV or shRNA‐SHP‐1‐rLV. The severity of inflammation was determined with biomicroscopy at 0, 24, 48, and 72 hours after LPS treatment. Consistent with a previous report,^13^ inflammation was most severe after 24 hours and returned to a near‐normal level at 72 hours. The knockdown of SHP‐1 exacerbated the severity of inflammation between 24 and 72 hours, compared with that in the blank‐rLV group (Figure 1E,F). Visual dysfunction determined by ERG was evident in eyes with EIU.^21^, ^22^ Therefore, we recorded the maximal ERG 24 hours after LPS induction, following dark adaptation for 12 hours. The amplitudes of the a and b waves were dampened in the blank‐rLV + LPS group compared with those in the control group. The amplitudes of these waves were further reduced in the shRNA‐SHP‐1‐rLV + LPS group (Figure 1G,H). These results indicate that SHP‐1 knockdown exacerbates visual dysfunction in EIU.

### 3.2 SHP‐1 knockdown exacerbates cell infiltration in the anterior chamber and vitreous body

**FIGURE 2:**
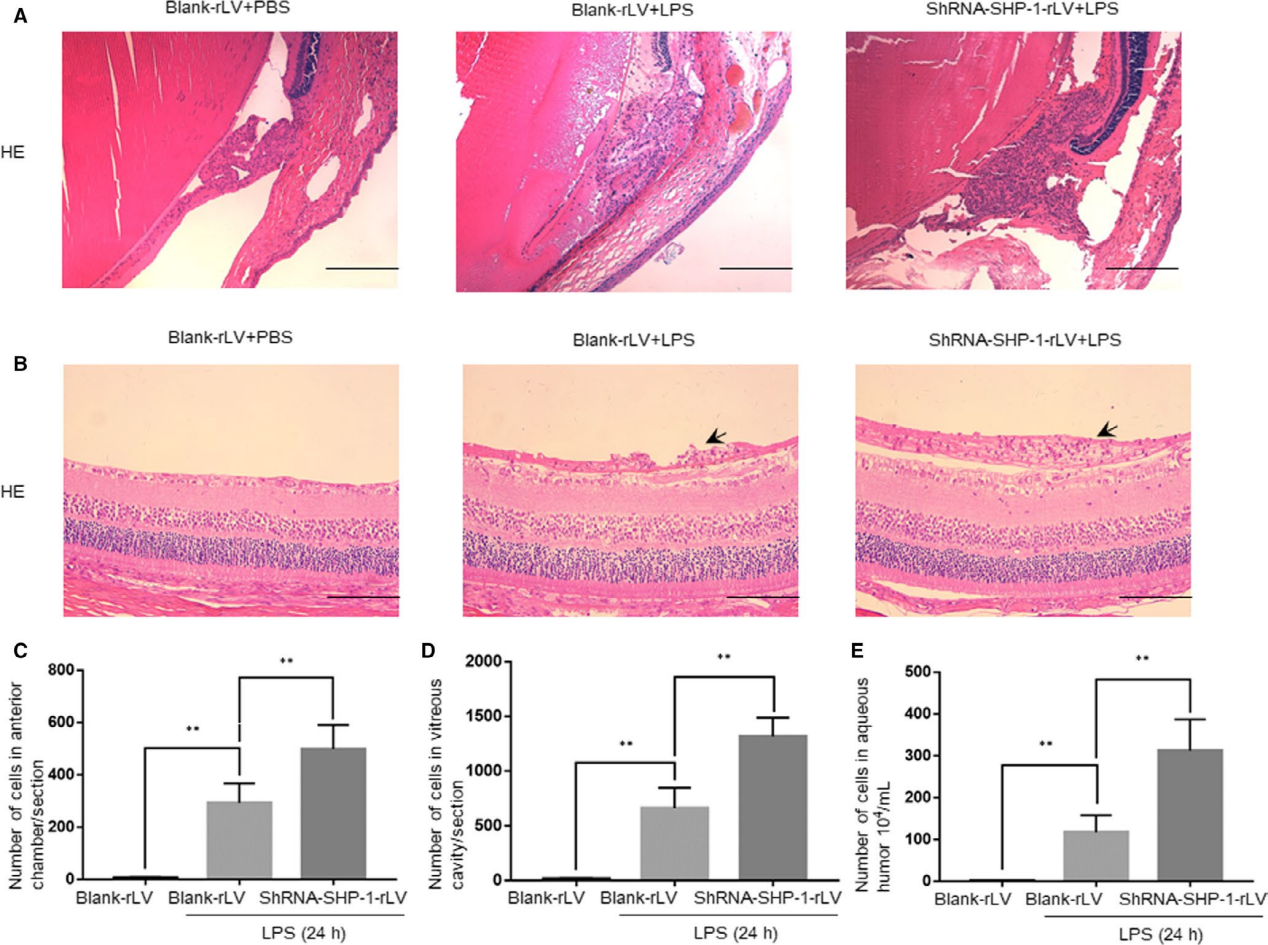
SHP‐1 knockdown exacerbates cellular infiltration in the anterior chamber and vitreous body. A, Representative images of the iris‐ciliary body in the blank‐rLV + PBS, blank‐rLV + LPS and shRNA‐SHP‐1‐rLV + LPS groups 24 h after LPS administration. Scale bar: 200 μm. B, Representative images of the border between the retina and vitreous body in the blank‐rLV + PBS, blank‐rLV + LPS and shRNA‐SHP‐1‐rLV + LPS groups at 24 h after LPS administration. Scale bar: 100 μm. C‐E, Quantitative analysis of the number of infiltrating cells in the anterior chamber per section (C), the number of infiltrating cells in the vitreous body per section (D) and the number of free cells in the aqueous humour (E). One‐way ANOVA followed by Dunnett's test was used. n = 5 per group. **P < .01

Histological examinations revealed no obvious cell infiltration in either the anterior chamber around the iris‐ciliary body or the vitreous body lining the inner limiting membrane in the blank‐rLV + PBS group. However, leucocytes accumulated at both of these sites in the blank‐rLV + LPS group, and to even greater degrees in the shRNA‐SHP‐1‐rLV + LPS group (Figure 2A,B). The numbers of infiltrating cells in the anterior chamber and vitreous body were about 1.7‐ and twofold higher, respectively, in the shRNA‐SHP‐1‐rLV + LPS group than in the blank‐rLV + LPS group (Figure 2C,D). SHP‐1 knockdown also increased the LPS‐induced free cell exudate in the aqueous humour (Figure 2E).

### 3.3 Knockdown of SHP‐1 aggravates the inflammatory responses in the retina and aqueous humour

**FIGURE 3:**
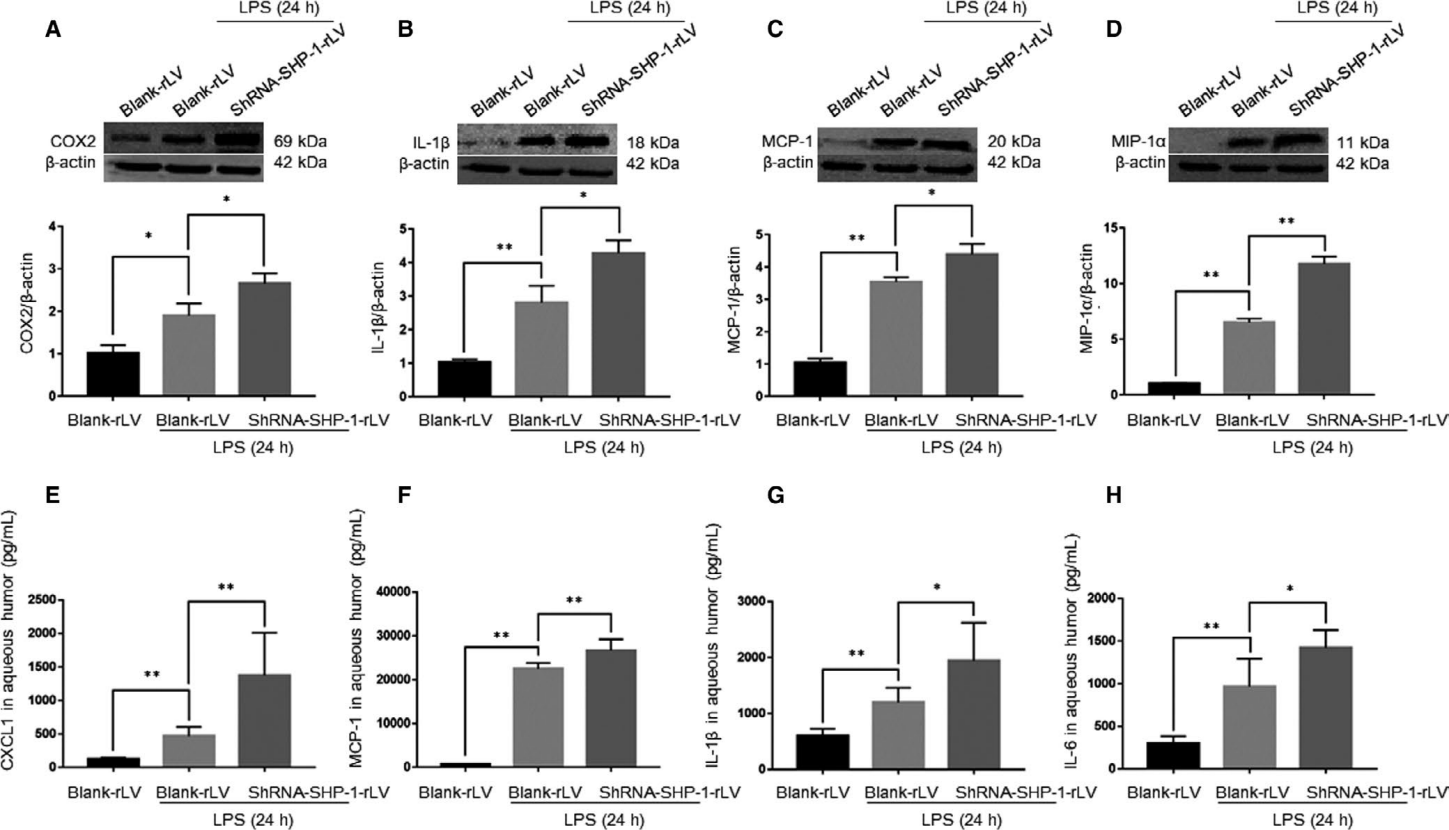
SHP‐1 knockdown increases the expression of inflammatory mediators in the retina and aqueous humour. A‐D, Expression levels of COX2 (A), IL‐1β (B), MCP‐1 (C) and MIP‐1α (D) at 24 h after intravitreal injection of LPS. One‐way ANOVA followed by Dunnett's test was used. n = 3 per group. E‐H, Concentrations of CXCL1 (E), MCP‐1 (F), IL‐1β (G) and IL‐6 (H) in the aqueous humour. One‐way ANOVA followed by Dunnett's test was used. n = 5 per group.*P < .05 and **P < .01

A variety of inflammatory mediators are expressed in the retina and secreted into the aqueous humour and may play pivotal roles in the development of ocular inflammation.^11^, ^23^, ^24^ Therefore, Western blotting and ELISAs were used to determine the roles of SHP‐1 in regulating inflammatory mediators in EIU. The retinal abundances of COX2, IL‐1β, MCP‐1 and MIP‐1α were higher in the blank‐rLV + LPS group than in the blank‐rLV + PBS group, and their expression was further increased by SHP‐1 knockdown (Figure 3A‐D). ELISAs of the aqueous humour samples revealed that SHP‐1 knockdown enhanced the LPS‐induced production of IL‐1β, CXCL1, MCP‐1 and IL‐6 (Figure 3E‐H). These results indicate that LPS‐induced inflammation in the retina was aggravated by SHP‐1 knockdown.

### 3.4 Overexpression of SHP‐1 alleviates the inflammatory response and cell infiltration

**FIGURE 4:**
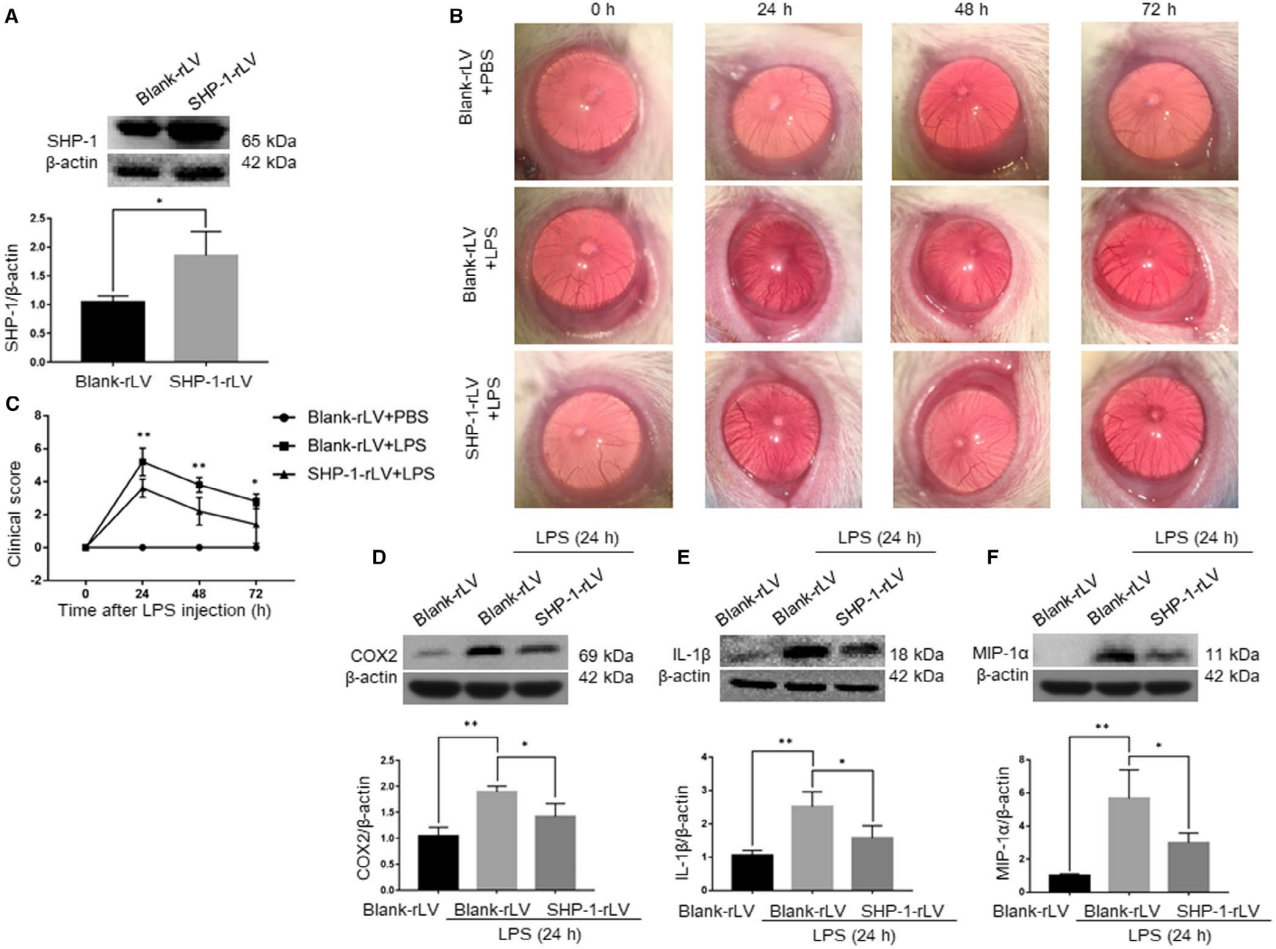
Overexpression of SHP‐1 down‐regulates the expression of inflammatory mediators and attenuates the inflammatory responses in the retina. A, Western blotting analysis of SHP‐1 expression 1 wk after intravitreal injection of blank‐rLV or SHP‐1‐rLV. Unpaired *t* test was used. n = 3 per group. B, Representative biomicroscopy images of the blank‐rLV + PBS, blank‐rLV + LPS and SHP‐1‐rLV + LPS groups at 0, 24, 48 and 72 h after LPS treatment. C, Comparison of clinical scores among the three groups. One‐way ANOVA followed by Dunnett's test was used. n = 5 per group. D‐F, Western blotting analysis of COX2 (D), IL‐1β (E) and MIP‐1α (F) expression in the retina at 24 h after LPS administration. One‐way ANOVA followed by Dunnett's test was used. n = 3 per group. *P < .05 and **P < .01

We also examined the effects of SHP‐1 overexpression on the LPS‐induced inflammatory response in the retina. SHP‐1 overexpression was induced by delivering SHP‐1‐rLV intravitreally. One week after SHP‐1‐rLV delivery, SHP‐1 expression was up‐regulated nearly twofold (Figure 4A). After LPS treatment, the severity of inflammation in the anterior segment was evaluated as described above. We found that inflammation was attenuated in the SHP‐1‐rLV + LPS group at 24 hours after LPS induction compared with that in the blank‐rLV + LPS group, and that SHP‐1 overexpression helped return to nearly normal state at 48 hours (Figure 4B,C). We also performed Western blotting to determine the protein expression levels of inflammatory mediators in the retina. The LPS‐induced increases in the expression of COX2, IL‐1β and MIP‐1α were significantly mitigated by the overexpression of SHP‐1 in the retina (Figure 4D‐F). These results show that the overexpression of SHP‐1 alleviates the inflammatory response in EIU.

**FIGURE 5:**
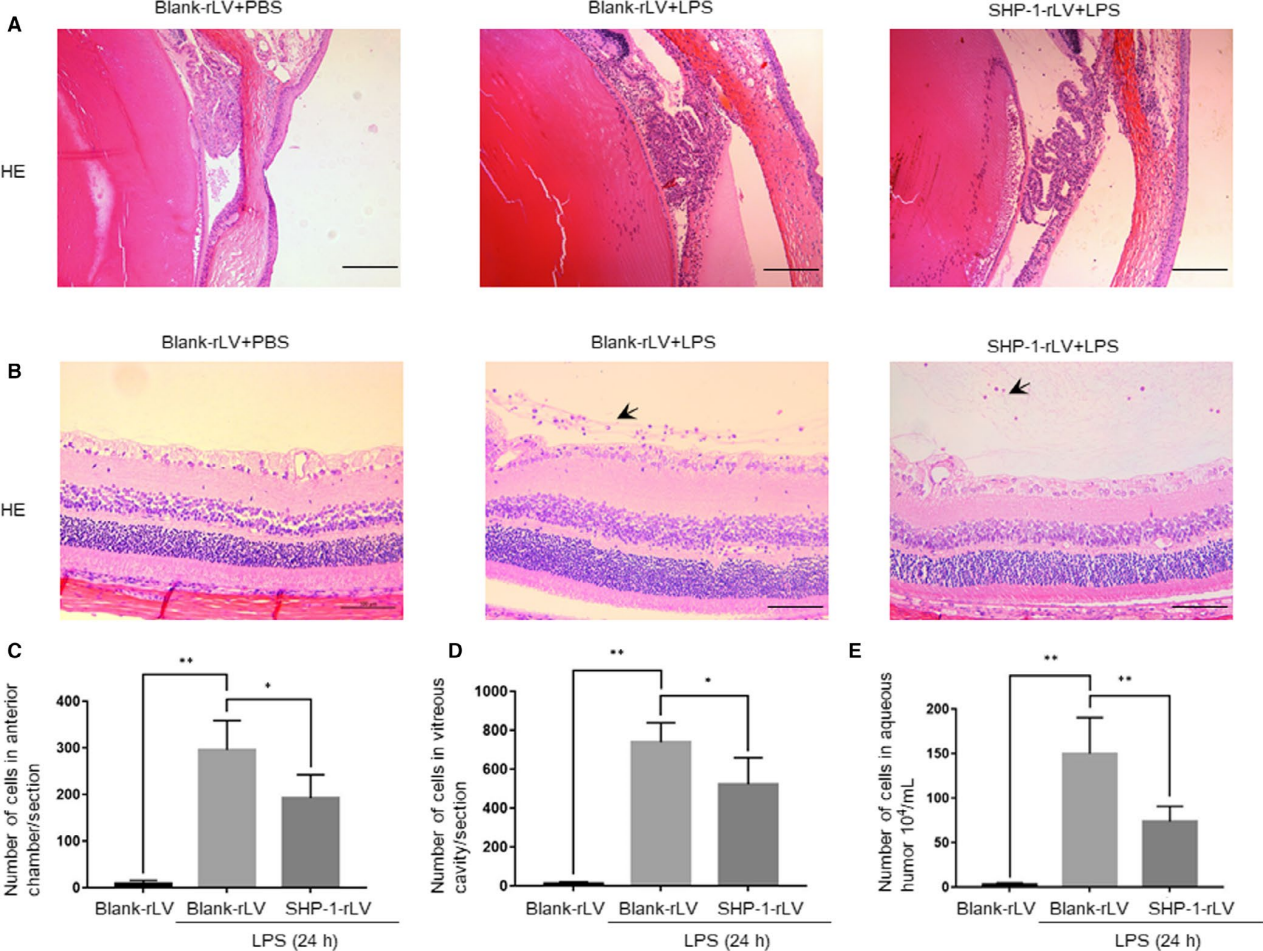
SHP‐1 overexpression reduces infiltrating cells into the anterior chamber and vitreous body. A, Representative images of the iris‐ciliary body in the blank‐rLV + PBS, blank‐rLV + LPS and SHP‐1‐rLV + LPS groups at 24 h after LPS treatment. Scale bar: 200 μm. B, Representative images of the border between the retina and vitreous body in the blank‐rLV + PBS, blank‐rLV + LPS and SHP‐1‐rLV + LPS groups at 24 h after LPS treatment. Scale bar: 100 μm. C‐E, Calculation of the number of infiltrating cells per section in the anterior chamber (C) and vitreous body (D), and the number of free cells in the aqueous humour (E). One‐way ANOVA followed by Dunnett's test was used. n = 5 per group. *P < .05 and **P < .01

We also performed H&E staining of the retinal sections. The results showed that fewer leucocytes were recruited into the anterior chamber and vitreous body in the SHP‐1‐rLV + LPS group than in the blank‐rLV + LPS group (Figure 5A,B). The total numbers of infiltrating cells in the anterior chamber, vitreous body and aqueous humour in the SHP‐1‐rLV + LPS group were equivalent to about 65%, 70% and 50% of those in the blank‐rLV + LPS group, respectively (Figure 5C‐E). These results indicate that the overexpression of SHP‐1 reduces infiltrating cells in both the anterior chamber and vitreous body.

### 3.5 SHP‐1 inhibits the expression of inflammatory cytokines in Müller cells

**FIGURE 6:**
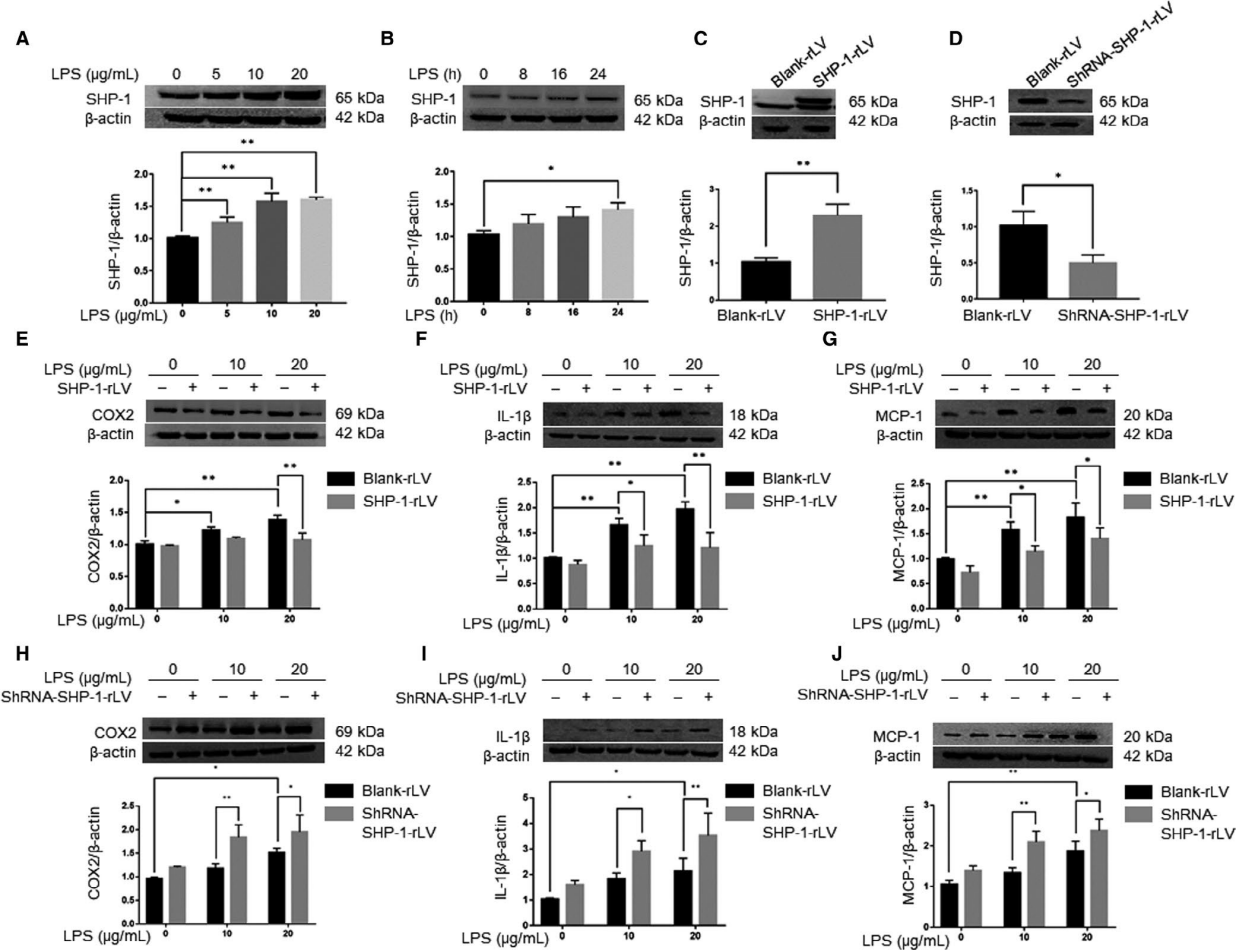
SHP‐1 regulates the inflammatory response to LPS treatment in rMC‐1 cells. A, Western blotting analysis of SHP‐1 protein expression in rMC‐1 cells incubated with 0, 5, 10 or 20 μg/mL LPS for 24 h. B, Western blotting analysis of SHP‐1 expression in rMC‐1 cells incubated with 10 μg/mL LPS for 0, 8, 16 or 24 h. One‐way ANOVA followed by Dunnett's test was used to compare the groups. n = 3 per group. *P < .05 and **P < .01. C, Overexpression efficiency of SHP‐1‐rLV confirmed by Western blotting. D, Western blotting showed that SHP‐1 was significantly reduced in rMC‐1 cells by shRNA‐SHP‐1‐rLV. E‐G, After stable transfection, the rMC‐1 cells were incubated with 10 μg/mL LPS for 24 h and Western blotting was performed to determine the protein abundances of COX2 (E), IL‐1β (F) and MCP‐1 (G). H‐J, After stable transfection, rMC‐1 cells were incubated with 10 μg/mL LPS for 24 h and Western blotting was performed to determine the protein abundances of COX2 (H), IL‐1β (I) and MCP‐1 (J). Two‐way ANOVA followed by Dunnett's test was used. n = 3 per group. *P < .05 and **P < .01

Müller cells are the major glial cells in the retina, and express Toll‐like receptors, especially TLR4, which act as receptors for LPS and contribute to the inflammation in EIU.^23^ Because SHP‐1 was significantly up‐regulated in Müller cells in EIU, we investigated the possible role of SHP‐1 in rMC‐1 in vitro. rMC‐1 cells were exposed to 0, 5, 10, and 20 µg/mL LPS for 24 hours, and Western blotting showed that SHP‐1 expression was increased in a concentration‐dependent manner (Figure 6A). Exposing rMC‐1 cells to 10 µg/mL LPS for 0, 8, 16 and 24 hours showed that SHP‐1 expression increased in a time‐dependent manner (Figure 6B). These data indicate that LPS induces SHP‐1 expression in a time‐ and concentration‐dependent manner.

We next transfected rMC‐1 cells with SHP‐1‐rLV to induce SHP‐1 overexpression and performed Western blotting to confirm the overexpression efficiency (Figure 6C). The expression of COX2, IL‐1β and MCP‐1 was up‐regulated in rMC‐1 cells transfected with blank‐rLV and exposed to LPS at 10 or 20 µg/mL. These changes were significantly inhibited by SHP‐1 overexpression (Figure 6E‐G). We then knocked down SHP‐1 in rMC‐1 cells to confirm its role in inflammation and verified the knockdown efficiency with Western blotting (Figure 6D). In this experiment, LPS increased the expression of the proinflammatory factors (COX2, IL‐1β and MCP‐1), which was further enhanced by SHP‐1 knockdown (Figure 6H‐J). These results suggest SHP‐1 acts as an acute reactive protein in response to an inflammatory agent (ie LPS) to alleviate the proinflammatory state and that the absence of SHP‐1 results in uncontrolled inflammation.

### 3.6 SHP‐1 down‐regulates the TAK1/JNK pathway in Müller cells in vitro

**FIGURE 7:**
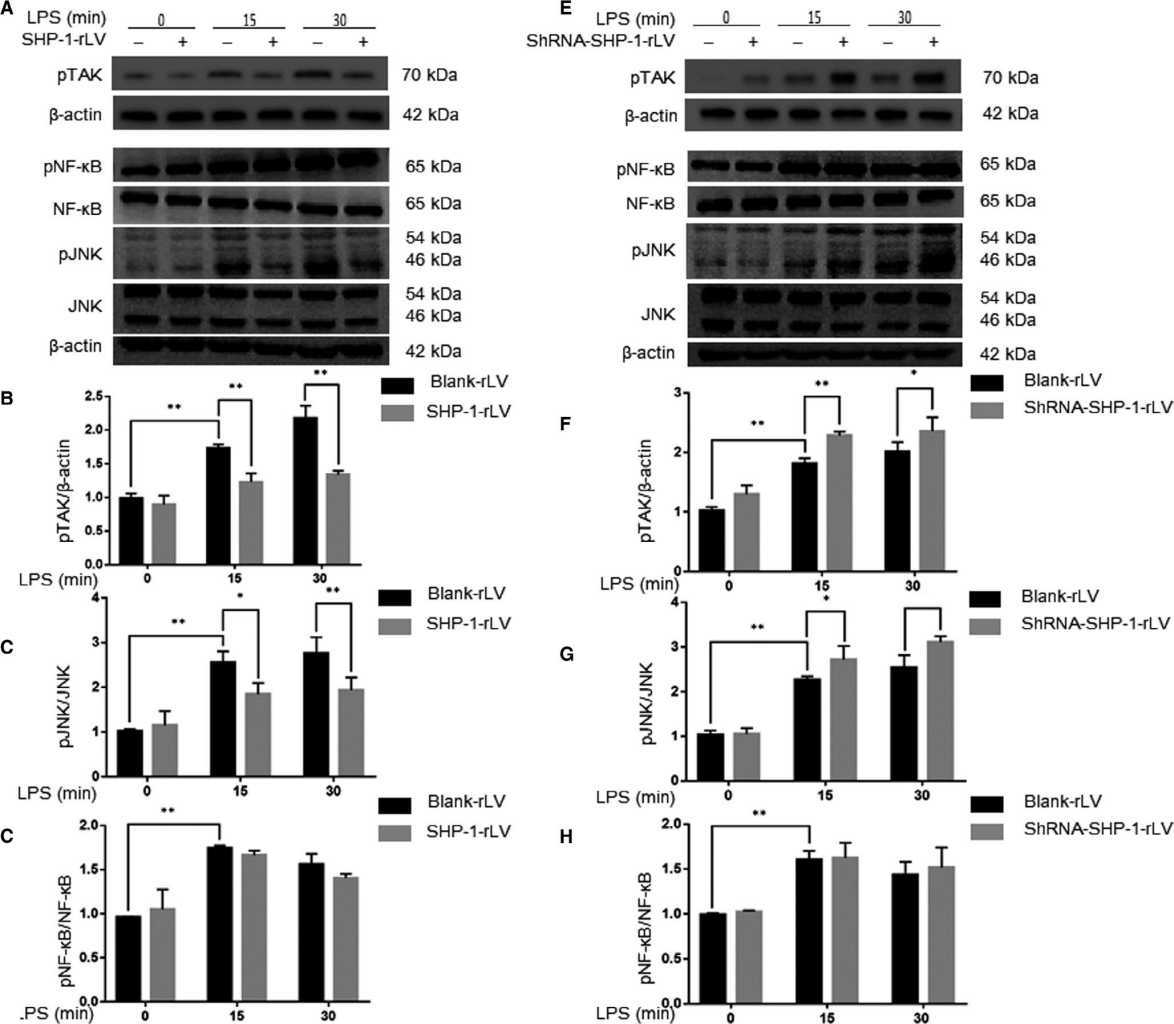
SHP‐1 negatively regulates kinase phosphorylation in LPS‐treated rMC‐1 cells. A, Effects of SHP‐1 overexpression on inflammatory signalling pathways. Blank‐rLV‐ and SHP‐1‐rLV‐ transfected rMC‐1 cells were exposed to 10 μg/mL LPS for 0, 15 or 30 min, and Western blotting was performed to determine the phosphorylation status of TAK1, JNK and NF‐κB and the total protein levels of JNK and NF‐κB. B‐D, Quantitative analysis of pTAK1/β‐actin (B), pJNK/JNK (C) and pNF‐κB/NF‐κB (D). E, Effects of SHP‐1 knockdown on inflammatory signalling pathways. Blank‐rLV‐ and shRNA‐SHP‐1‐rLV‐transfected rMC‐1 cells were exposed to 10 μg/mL LPS for 0, 15, or 30 min, and Western blotting was performed to determine the phosphorylation status of TAK1, JNK and NF‐κB and the total protein levels of JNK and NF‐κB. F‐H, Quantitative analysis of pTAK1/β‐actin (F), pJNK/JNK (G) and pNF‐κB/NF‐κB (H). Two‐way ANOVA followed by Dunnett's test was used. n = 3 per group. *P < .05 and **P < .01

TAK1, a member of the mitogen‐activated protein kinase kinase kinase (MAPKKK) family, is reported to be a direct downstream target of SHP‐1 and is implicated in various inflammatory signalling pathways.^25^ Downstream of TAK1 mainly lie the NF‐κB pathway and JNK pathway, which play important roles in inflammation.^26^ To clarify the signalling pathways by which SHP‐1 regulates the inflammatory response in Müller cells, we examined the phosphorylation of TAK1, JNK and NF‐κB with Western blotting. Exposure to LPS increased the phosphorylation status of TAK1, NF‐κB and JNK. Notably, SHP‐1 overexpression significantly attenuated the phosphorylation of TAK1 and JNK, but not that of NF‐κB (Figure 7A‐D). Conversely, SHP‐1 knockdown significantly increased TAK1 and JNK phosphorylation, but not NF‐κB phosphorylation, in Müller cells (Figure 7E‐H). These results imply that SHP‐1 regulates the production of inflammatory mediators via the phosphorylation of TAK1 and JNK, but not that of NF‐κB.

### 3.7 Inhibition of the JNK pathway attenuates LPS‐induced inflammation in the retina

**FIGURE 8:**
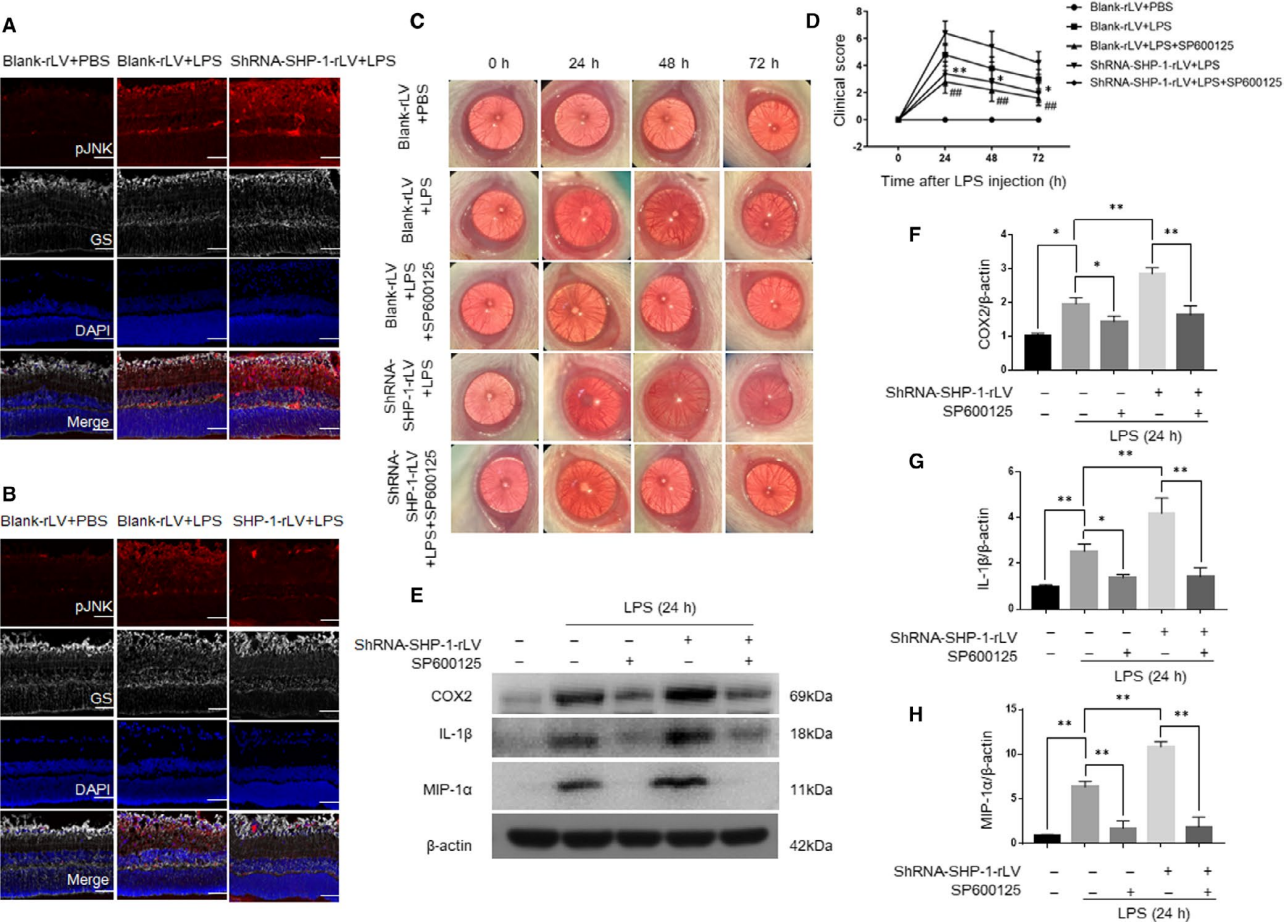
Inflammation induced by LPS was mitigated by the inhibition of the JNK pathway in the retina. A, Representative immunofluorescent images of the blank‐rLV + PBS, blank‐rLV + LPS and shRNA‐SHP‐1‐rLV + LPS groups at 24 h after LPS administration. B, Representative images of the blank‐rLV + PBS, blank‐rLV + LPS and SHP‐1‐rLV + LPS groups at 24 h after LPS administration. Red: pJNK; grey: GS; blue: DAPI. Scale bar: 50 μm. C, Representative biomicroscopic images of the blank‐rLV + PBS, blank‐rLV + LPS, blank‐rLV + LPS+SP600125, shRNA‐SHP‐1‐rLV + LPS, shRNA‐SHP‐1‐rLV + LPS+SP600125 groups at 0, 24, 48 and 72 h after LPS treatment. D, Clinical scores for the five groups described above. One‐way ANOVA followed by Dunnett's test was used. n = 5 per group.*: blank‐rLV + LPS+SP600125 group vs blank‐rLV + LPS group; #: shRNA‐SHP‐1‐rLV + LPS+SP600125 group vs shRNA‐SHP‐1‐rLV + LPS group. E‐H, Western blotting analysis of the retinal expression of COX2, IL‐1β and MIP‐1α at 24 h after LPS administration. One‐way ANOVA followed by Dunnett's test was used. n = 3 per group. */^#^ *P* < .05 and **/^# #^ *P* < .01

We next examined the role of the SHP‐1‐regulated TAK1/JNK pathway in LPS‐induced inflammation in vivo. A Western blotting analysis of the retina at 0, 6, 12 and 24 hours after the intravitreal injection of LPS showed that the phosphorylation of TAK1 and JNK in the retina was increased by LPS treatment (Figure S1A–C). As shown in Figure 8A,B, co‐staining for p‐JNK and GS indicated that the expression of p‐JNK in the Müller cells was significantly enhanced by LPS treatment in vivo. The knockdown of SHP‐1 further strengthened JNK phosphorylation during LPS treatment, whereas SHP‐1 overexpression attenuated the LPS‐induced phosphorylation of JNK (Figure 8A,B). SP600125 (a JNK inhibitor) was used in this study to investigate whether the effects of SHP‐1 knockdown on EIU were mediated by JNK activation. As evident in biomicroscopic images and clinical scores, inhibition of the JNK pathway reduced the severity of inflammation after LPS treatment and abrogated the intensifying effect of SHP‐1 knockdown (Figure 8C,D). Western blotting showed that SHP‐1 knockdown exacerbated LPS‐induced expression of COX2, IL‐1β and MIP‐1α in the retina, which was abrogated by SP600125 (Figure 8E‐H). These results indicate that the JNK pathway, which is negatively regulated by SHP‐1, participates in LPS‐induced inflammation in the retina.

## 4 DISCUSSION

SHP‐1 belongs to the protein‐tyrosine phosphatase non‐receptor family and plays important roles in regulating inflammatory signalling pathways. It is recruited to cytoplasmic immunoreceptor tyrosine‐based inhibition motifs (ITIMs) and dephosphorylates activated proteins.^27^ Previous studies have shown that SHP‐1 regulates the responsiveness of macrophages to chemokines and their infiltration of infectious sites,^28^ and dampens the LPS‐mediated up‐regulation of TNF‐α and inducible nitric oxide synthase (iNOS).^29^ Interestingly, SHP‐1 is also targeted by pathogens, including *Leishmania*, to prevent inflammatory cell activation and to subvert the host's immune response.^30^ However, until now, it has been unclear whether SHP‐1 is involved in the development of uveitis. In the present study, we found that SHP‐1 is up‐regulated in response to LPS, attenuates LPS‐induced production of inflammatory mediators and improves retinal function by suppressing the activation of the TAK1/JNK pathway.

An important finding of our study is that LPS‐induced SHP‐1 negatively regulates inflammation in the retina. Although the inhibitory effects of SHP‐1 on inflammation have been reported, the expression of SHP‐1 in different tissues after stimulation with various agents is not always consistent. Growing evidence indicates that SHP‐1 is up‐regulated in response to some stressors, and acts as an autoinhibitory molecule that suppresses excessive inflammation. For example, SHP‐1 was up‐regulated in acute pancreatitis, independently of neutrophil infiltration, and in astrocytes after focal cerebral ischaemia.^7^, ^31^ It has been reported that TNF‐α increases SHP‐1 activity and expression in cultured endothelial cells in vitro. ^32^ Consistent with these studies, our results show that SHP‐1 expression is increased in the retina of rats with EIU and in LPS‐treated Müller cells. We consider that SHP‐1 is primarily up‐regulated in the resident retinal Müller cells, as a means of self‐protection and contributes to the restoration of retinal homoeostasis. The SHP‐1 knockdown reportedly weakens the ability of cells to inhibit the signals activated by various stimuli.^33^ More intense cellular recruitment is observed at the infection sites in viable motheaten SHP‐1‐deficient mice.^34^ Notably, in our EIU model, the inflammation of the anterior segment tended to self‐restrict at 72 hours after the administration of LPS. SHP‐1 knockdown caused a more notable inflammation peak 24 hours after LPS induction, and a slower resolution rate over a period of 72 hours. Conversely, SHP‐1 overexpression mitigated the LPS‐induced inflammatory response in the anterior segment. Consistent with these clinical findings, SHP‐1 knockdown exacerbated cell infiltration, whereas SHP‐1 overexpression elicited opposite effects. Taken together, the up‐regulated SHP‐1 in the retina acts as an important anti‐inflammatory effector in EIU.

Chemokines, such as MCP‐1, MIP‐1α and CXCL1, are responsible for neutrophil recruitment during inflammation and are critical mediators of ocular inflammation.^35^, ^36^, ^37^ It has been reported that MCP‐1 knockout alleviates the infiltration of inflammatory cells into the eye.^38^ The chemokines produced by resident cells are responsible for the initiation of subsequent cell infiltration.^39^ COX2 is another key modulator of inflammation.^40^ COX2 expression in retinal microglia marks their polarization to a proinflammatory phenotype,^41^ and the suppression of COX2 and the production of prostaglandin E_2_ alleviates the inflammation in EIU.^42^ The knockdown or inhibition of SHP‐1 increases the production of TNF‐α, iNOS, and IL‐1β in macrophages, microglia and dendritic cells exposed to LPS.^16^, ^29^, ^43^ To determine the role of SHP‐1 in LPS‐induced inflammation, we examined whether these inflammatory mediators are regulated by SHP‐1. Our results show that the LPS‐ induced expression of MCP‐1, MIP‐1α and CXCL1 was increased by SHP‐1 knockdown in the retina. The synthesis of IL‐1β and IL‐6 was also increased by SHP‐1 knockdown. By contrast, SHP‐1 overexpression attenuated the effects of LPS on these inflammatory mediators. Similar results were obtained in LPS‐treated Müller cells. These results indicate that SHP‐1 inhibits the expression of inflammatory mediators during LPS‐induced inflammation in the retina. Chemokines and proinflammatory cytokines also contribute to tissue injury. MCP‐1 produced by Müller cells is a key mediator of photoreceptor apoptosis during retinal detachment,^44^ and IL‐1β is reported to promote the release of reactive oxygen species in the retinal pigment epithelium.^45^ In our study, the results of ERG indicate that the LPS‐induced inflammation injured photoreceptors, resulting in retinal dysfunction, which was aggravated by SHP‐1 knockdown. Therefore, up‐regulation of SHP‐1 may counterbalance the responses to various stimuli to avoid the harmful side effects of the inflammatory response. These findings provide compelling evidence that SHP‐1 inhibits the expression of inflammatory mediators and protects against visual dysfunction during LPS‐induced inflammation in the retina.

The modulatory effects of SHP‐1 on inflammation are mostly dependent on the dephosphorylation of the kinases involved in the inflammatory pathways, especially the NF‐κB and MAPK pathways.^14^ Mice with point mutations in the gene encoding SHP‐1 develop an inflammatory skin disease, characterized by the spontaneous activation of TAK1.^46^ TAK1 activates JNK in response to various stressors, including LPS.^47^, ^48^ SHP‐1 is also reported to maintain TAK1/JNK/AP‐1 activity in U937 cells.^49^ Therefore, we investigated whether the TAK1/JNK and NF‐κB pathways are involved in the SHP‐1‐regulated inflammatory response in the retina. Our results show that the phosphorylation of TAK1 and JNK in response to LPS is negatively regulated by SHP‐1, whereas NF‐κB activation is not modulated by SHP‐1. These findings are consistent with those of a previous study in which the absence of SHP‐1 in macrophages increased the phosphorylation of JNK but not that of NF‐κB.^50^ Moreover, we also found that the TAK1/JNK pathway is activated in EIU and that the inhibition of JNK activation with SP600125 weakened the enhancing effects of SHP‐1 knockdown on the LPS‐induced inflammatory response. These findings demonstrate that SHP‐1 controls the synthesis of LPS‐induced proinflammatory mediators, at least in part, via the TAK1/JNK pathway. Although we have demonstrated the important roles of SHP‐1 on LPS‐induced inflammation in the retina, the intravitreal administration of a lentivirus may alter SHP‐1 expression in a variety of cell types, including Müller cells. Therefore, vectors that are more specific and can alter the expression of SHP‐1 or other genes in specific cell types should be used in future studies.

In summary, our results imply that SHP‐1 plays an essential role in the pathogenesis of EIU and in LPS‐induced inflammation in rMC‐1 cells. SHP‐1 mitigates inflammation by suppressing the activity of the TAK1/JNK pathway. This work provides direct evidence of the effects of SHP‐1 on the development and resolution of uveitis and suggests that SHP‐1 may offer a potential target for the management of ocular inflammatory diseases in the future.

## CONFLICT OF INTEREST

The authors declare no conflict of interest associated with this manuscript.

## AUTHOR CONTRIBUTION

**Gezhi Xu:** Funding acquisition (lead); Supervision (lead). **Xiaonan Zhuang:** Conceptualization (lead); Data curation (lead); Formal analysis (lead); Investigation (lead); Methodology (lead); Writing‐original draft (lead); Writing‐review & editing (lead). **Jun Ma:** Data curation (lead); Investigation (lead); Resources (equal); Writing‐original draft (equal); Writing‐review & editing (lead). **Sisi Xu:** Data curation (equal); Investigation (lead); Methodology (lead). **Zhongcui Sun:** Formal analysis (equal); Investigation (equal); Methodology (equal); Resources (equal); Validation (equal). **Rong Zhang:** Investigation (equal); Methodology (equal). **Meng Zhang:** Investigation (equal); Methodology (equal).

## Supporting information

Fig S1

## Data Availability

The data that support the findings of this study are available from the corresponding author upon reasonable request.
