# Corrigendum

**DOI:** 10.1111/jcmm.17446

**Published:** 2022-07-06

In Xiaonan Zhuang et al.^1^, the picture of ShRNA‐SHP‐1‐rLV + LPS + SP600125 group in Figure 8C is incorrect. The corrected Figure 8 is shown below. The authors confirm that all the results and conclusions of this article remain unchanged.

**FIGURE 8:**
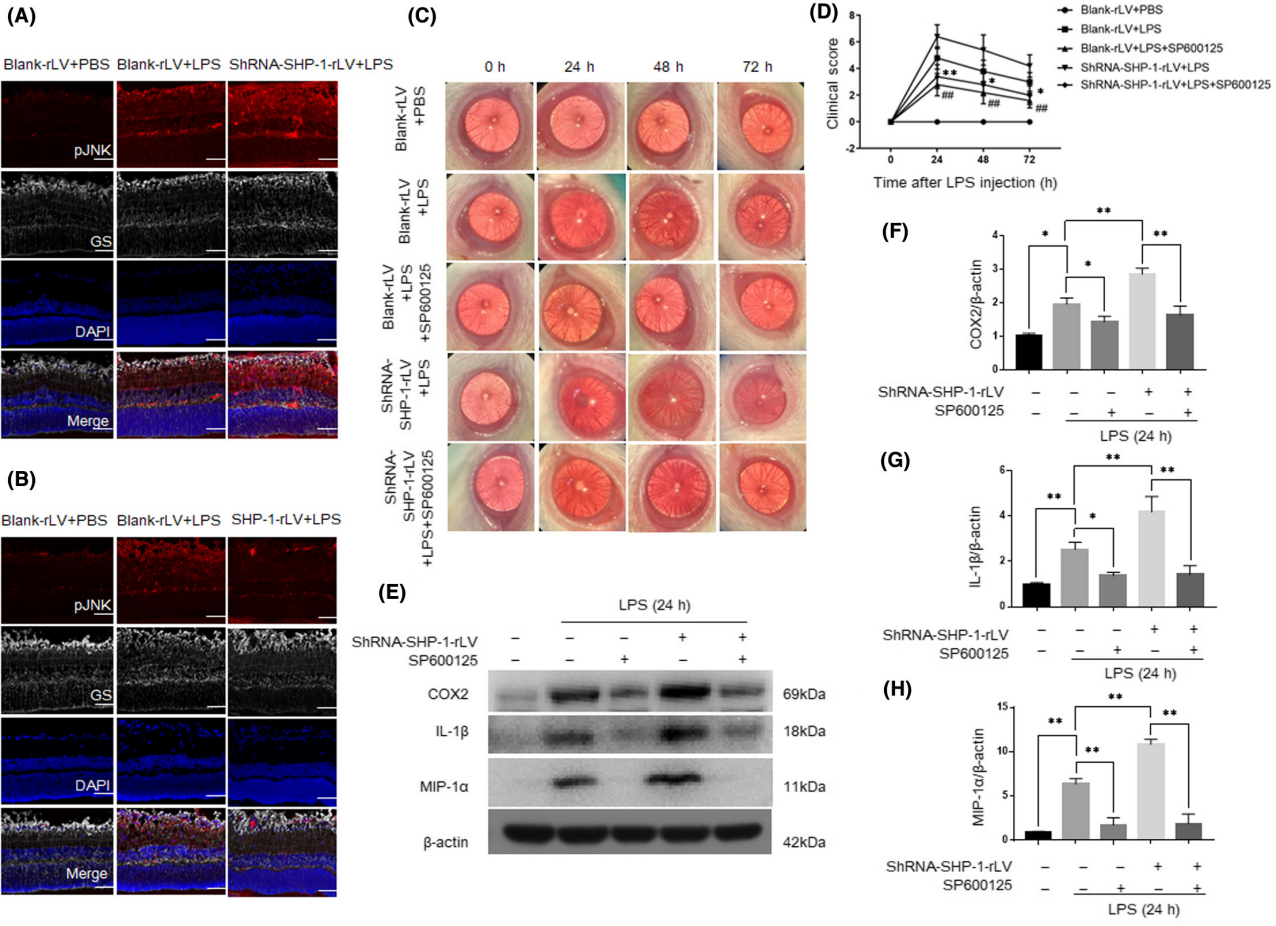
Inflammation induced by LPS was mitigated by the inhibition of the JNK pathway in the retina. (A) Representative immunofluorescent images of the blank‐rLV + PBS, blank‐rLV + LPS and shRNA‐SHP‐1‐rLV + LPS groups at 24 h after LPS administration. (B) Representative images of the blank‐rLV + PBS, blank‐rLV + LPS and SHP‐1‐rLV + LPS groups at 24 h after LPS administration. Red: pJNK; grey: GS; blue: DAPI. Scale bar: 50 μm. (C) Representative biomicroscopic images of the blank‐rLV + PBS, blank‐rLV + LPS, blank‐rLV + LPS + SP600125, shRNA‐SHP‐1‐rLV + LPS, shRNA‐SHP‐1‐rLV + LPS + SP600125 groups at 0, 24, 48 and 72 h after LPS treatment. (D) Clinical scores for the five groups described above. One‐way anova followed by Dunnett's test was used. *n* = 5 per group. *Blank‐rLV + LPS + SP600125 group versus blank‐rLV + LPS group; ^#^shRNA‐SHP‐1‐rLV + LPS + SP600125 group versus shRNA‐SHP‐1‐rLV + LPS group. (E–H) Western blotting analysis of the retinal expression of COX2, IL‐1β and MIP‐1α at 24 h after LPS administration. One‐way anova followed by Dunnett's test was used. *n* = 3 per group. ^*/ #^ *p* < 0.05 and ^**/# #^ *p* < 0.01
