# Supplementary material for: SHP‐1 suppresses endotoxin‐induced uveitis by inhibiting the TAK1/JNK pathway
Source: J Cell Mol Med. 2020 Nov 18;25(1):147–60. doi: 10.1111/jcmm.15888 (PMC7810969; doi:10.1111/jcmm.15888)
Supplement: Supplementary file 1 — Fig S1 [file JCMM-25-147-s001.docx]

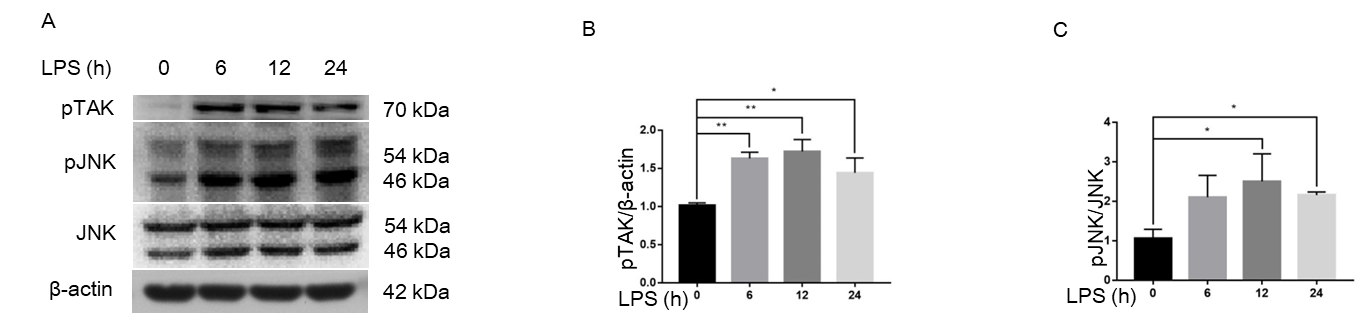


Fig. S1. TAK1/JNK pathway is activated in EIU. (A) TAK1/JNK phosphorylation of retina 0h, 6h, 12h and 24h after LPS administration. The phosphorylated levels of TAK1(B) and JNK(C) were determined by western analysis. One-way ANOVA followed by Dunnett’s test was used. *n* = 3 per group. *P< 0.05 and **P< 0.01.
